# Recurrent de novo variants in the spliceosomal factor *CRNKL1* are associated with severe microcephaly and pontocerebellar hypoplasia with seizures

**DOI:** 10.1016/j.ajhg.2025.05.013

**Published:** 2025-06-18

**Authors:** Sankalita Ray Das, Rosie Sullivan, Mischa S G Ruegg, Julia Horsfield, Jordan Doran, Gemma Poke, Nathalie de Vries, Sarah Duerinckx, Damien Lederer, Muzhirah Haniffa, Wee-Teik Keng, Gaik-Siew Ch’ng, David A Parry, Andrew P Jackson, Masamune Sakamoto, Naomichi Matsumoto, Noriko Miyake, Shin Nabatame, Hidetoshi Taniguchi, Emma Wakeling, Deciphering Developmental Disorders Study, Katrin Õunap, Pilvi Ilves, Ghayda Mirzaa, Andrew Timms, Emily Pao, Kimberly A Aldinger, William Dobyns, Axel Bohring, Beate Behre, Daniel G Calame, James R Lupski, Juan M Pascual, Marc Abramowicz, Gregory Gimenez, Louise S Bicknell

**Affiliations:** 1 Department of Biochemistry, University of Otago, Dunedin, New Zealand; 2 Department of Pathology, Dunedin School of Medicine, University of Otago, Dunedin, New Zealand; 3 Central Hub, Genetic Health Service New Zealand, Te Whatu Ora - Health New Zealand, Wellington, New Zealand; 4 Health New Zealand, Department of Paediatrics, Palmerston North, New Zealand; 5 Department of Pediatric Neurology, Hôpital Universitaire de Bruxelles, Hôpital Erasme, Université Libre de Bruxelles, Brussels, Belgium; 6 Centre de Génétique Humaine, IPG, Charleroi, Belgique; 7 Department of Genetics, Hospital Kuala Lumpur, Kuala Lumpur, Malaysia; 8 Department of Genetics, Penang Hospital, Penang, Malaysia; 9 MRC Human Genetics Unit, Institute of Genetics and Cancer, University of Edinburgh, Edinburgh, UK; 10 Department of Human Genetics, Yokohama City University Graduate School of Medicine, Yokohama, Japan; 11 Department of Human Genetics, Research Institute, National Center for Global Health and Medicine, Tokyo, Japan; 12 Department of Pediatrics, the University of Osaka Graduate School of Medicine, Osaka, Japan; 13 North East Thames Regional Genetics Service, Great Ormond Street Hospital for Children NHS Foundation Trust, London, UK; 14 Genetics and Personalized Medicine Clinic, Tartu University Hospital, Tartu, Estonia; 15 Institute of Clinical Medicine, University of Tartu, Tartu, Estonia; 16 Radiology Clinic, Tartu University Hospital, Tartu, Estonia; 17 Department of Pediatrics, University of Washington School of Medicine, Seattle, WA, USA; 18 Brotman Baty Institute for Precision Medicine, Seattle, WA, USA; 19 Norcliffe Center for Integrative Brain Research, Seattle Children’s Research Institute, Seattle, WA, USA; 20 Department of Pediatrics, University of Washington, Seattle, WA, USA; 21 Department of Neurology, University of Washington, Seattle, WA, USA; 22 Department of Pediatrics, Division of Genetics and Metabolism, University of Minnesota, Minneapolis, MN, USA; 23 Centre of Medical Genetics, Department of Medical Genetics, University and University Hospital Münster, Münster, Germany; 24 Amedes MVZ for Pathology, Cytodiagnostics and Human Genetics, Halle/S., Germany; 25 Section of Pediatric Neurology and Developmental Neurosciences, Department of Pediatrics, Houston, TX, USA; 26 Texas Children’s Hospital, Houston, TX, USA; 27 Department of Molecular and Human Genetics, Baylor College of Medicine, Houston, TX, USA; 28 Human Genome Sequencing Center, Baylor College of Medicine, Houston, TX, USA; 29 Department of Pediatrics, Baylor College of Medicine, Houston, TX, USA; 30 Division of Child Neurology, Weill Cornell Medicine, Cornell University, New York, NY, USA; 31 Department of Genetic Medicine and Development, Faculty of Medicine, Université de Geneve, Geneva, Switzerland

## Abstract

Splicing is a complex process, required to create the transcriptomic diversity needed for specialised functions in higher eukaryotes. The spliceosome contains more than 100 proteins and RNA molecules which coordinate this dynamic process. Despite the ubiquity of splicing, pathogenic variants in spliceosomal components often cause a tissue-specific phenotype, hinting at further complexities which are not yet fully understood. We have identified a cohort of ten families with *de novo* missense variants in a spliceosomal component, CRNKL1, where nine individuals harbour one of two missense variants that both affect the same amino acid, p.Arg267. All affected individuals share a common and specific phenotype: profound pre- and post-natal microcephaly, with pontocerebellar hypoplasia, seizures and severe intellectual disability. Microinjection of mRNA encoding mutant Crnkl1 into a zebrafish model caused severe lack of brain development accompanied by a significant reduction in proliferating cells and widespread cellular stress, as indicated by p53 staining. RNA-seq analysis of injected zebrafish embryos showed broad transcriptomic changes, with altered expression of neuronal and cell cycle genes. Together, we have identified *CRNKL1* as a disease-associated gene and demonstrate the requirement for this protein in brain development. Our findings contribute to a growing disease cluster, where associated components act at the same spliceosomal stage and cause a severe neurological phenotype, suggesting a more intricate role for these spliceosomal subcomplexes than previously thought.

## Main Text

Splicing is an essential process which removes introns from pre-mRNA and creates mRNA diversity through alternative splicing to produce different transcript isoforms, particularly in the brain ^1,2^. The major spliceosome undertakes the majority of splicing in the cell. It is a massive complex containing over 100 protein and RNA species, many of which are evolutionarily conserved across eukaryotes to yeast ^3^. The major spliceosome is a dynamic complex, with protein subcomplexes and individual proteins joining and leaving the complex during the different stages of splicing. These dynamic interactions support both precise movement and reorganisation within the spliceosome to achieve accurate and timely intron removal. They can also function as part of a range of regulatory processes in the splicing of pre-mRNA transcripts to exert precise control on transcription maturation and progression to translation^3^.

The *CRNKL1* gene (MIM: 610952) encodes CRNKL1, also known as SYF3, a protein component of the spliceosome. CRNKL1 joins the complex during the ‘early B^act^ maturation state’, where the spliceosomal complex has been structurally rearranged to be activated but has not been catalytically primed by the RNA helicase PRP2, and so splicing of the RNA has not yet been initiated ^4^. The CRNKL1 protein joins independently and plays roles in supporting binding and disassociation as various proteins such as AQR and members of the intron-binding complex rearrange into their final positions ^4^. While the intricacies of the spliceosome are continuing to be uncovered, little attention has focused on the exact role of CRNKL1.

Spliceosomopathies are a group of disorders characterized by dysfunction of the spliceosome. Despite this complex being ubiquitous, many disorders exhibit a tissue-specific phenotype. Some dynamically associated proteins might be more enriched in certain tissues, or inaccurate splicing affects a specific transcript required by cells in that tissue, to cause the specific phenotypes, but for other disorders, the tissue-specific mechanisms are not yet clear ^5^. Disruption of splicing or alternative splicing is linked to several neurological disorders, including microcephalic osteodysplastic primordial dwarfism type 1 (MIM: 210710), ReNU syndrome (MIM: 620851), and autism spectrum disorder ^6–9^. Recently, Chai et al found that biallelic variants in *PPIL1* (MIM: 601301)and *PRP17* (MIM: 605585) cause similar neurodegenerative disorders, where affected individuals have microcephaly and pontocerebellar hypoplasia (MIM: 619301, 619302) ^10^. PPIL1 and PRP17 localise near CRNKL1 in the spliceosome. While PPIL1 and PRP17 interact in an isomerization reaction, this function does not seem altered in the presence of pathogenic variants, and so the precise consequence to the spliceosome remains unclear ^10^. In a mouse knock-in model of a human *PPIL1* variant, significant disruption to splicing was observed, with pathways associated with brain development or neurological disorders particularly affected ^10^. Many other forms of pontocerebellar hypoplasia have been associated with different types of RNA processing, potentially linking the spliceosome with this phenotype, but microcephaly is not such a consistent feature in these other forms ^11^.

Through exome sequencing and collaborations through Genematcher ^12^, we have identified ten individuals with recurrent *de novo* missense variants in *CRNKL1*. Two amino acid residues are affected by these variants, which appear to act structurally to support protein-protein binding and the active site configuration within the spliceosome. Introducing the variants into zebrafish through injection of mRNA caused severe disruption to organism development, with ventralisation and only minimal brain development. At the cellular level, the *crnkl1* mutant mRNA triggered significant cellular stress, apoptosis and failure of proliferation in the brain, with RNA-seq indicating widespread dysregulation of gene expression.

Together, our genetic and developmental functional results confirm variants in *CRNKL1* are associated with severe microcephaly, pontocerebellar hypoplasia and seizures. Our findings add to the range of conditions associated with the spliceosome, and together with the recent findings implicating variants in *PPIL1* and *PRP17* as causing a similar set of features, suggest a hotspot of spliceosomal proteins intimately involved in brain development.

**Figure 1:**
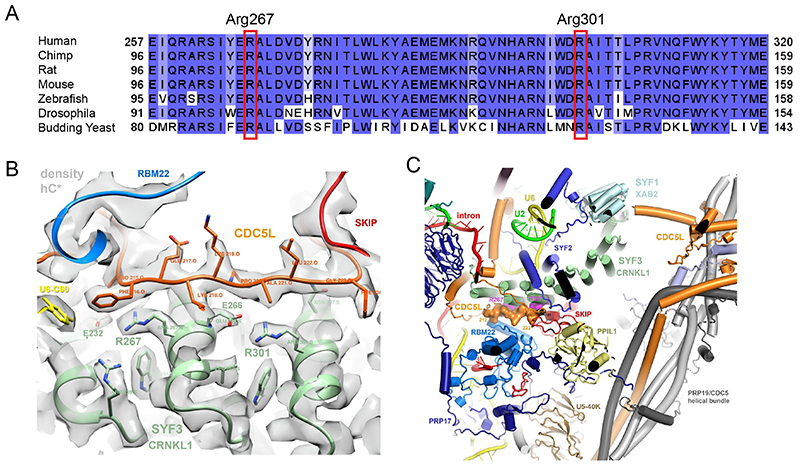
Residues Arg267 and Arg301 are fully conserved and lie in adjacent regions in CRNKL1 to support structural fidelity in the spliceosome. **A**. Clustal Omega alignment of protein sequences from a diverse range of organisms illustrates that both Arg267 and Arg301 are fully conserved throughout evolution. **B**. Within the spliceosome, Arg267 and Arg301 lie in adjacent α-helices, likely forming strong ionic interactions with neighbouring glutamic acids to help form a platform for CDC5L and to maintain structural integrity for the nearby U6 RNA active site. PBD: 8C6J. **C**. CRNKL1/SYF3 lies in close proximity to PPIL1 and PRP17 and likely binds initially and then supports the subsequent binding of a complex including PPIL1, U5-40K, RBM22, SKIP and PRP17.

As part of a research programme investigating genetic causes of microcephaly (ethical approval: New Zealand Health and Disability Ethics Committee: 16/STH/3), an affected European New Zealand individual with severe progressive microcephaly, pontocerebellar hypoplasia, severe intellectual disability, and seizures, and unaffected parents were analysed by trio exome sequencing (Agilent SureSelect Human All Exon V5 kit, Illumina Novaseq6000 150 bp paired-end sequencing). Genetic data was analysed using an adapted GATK pipeline and variants were prioritised under different inheritance models ^13^. Analysis of *de novo* variants revealed a missense variant in *CRNKL1*: c.800G>A, p.Arg267His (NM_016652.6), with segregation confirmed by Sanger sequencing. This variant is absent from gnomAD v4.1 and Arg267 is conserved throughout eukaryotes (Fig. 1A). Additional families were ascertained through international collaborations, Genematcher ^12^ and DECIPHER ^14^. A total of ten individuals have now been identified with *de novo* or heterozygous variants in *CRNKL1* (Table 1). Remarkably, nine of the ten individuals share one of two variants, either c.800G>A, or the immediately adjacent upstream nucleotide, c.799C>T, both of which impact the same residue, Arg267. The remaining individual, P10, has a different *de novo* variant, c.901C>G, p.Arg301Gly (NM_016652.6), located 34 amino acids downstream.

CRNKL1 is a subunit of the major spliceosome where it is commonly referred to as SYF3. The protein is composed of 16 tetratricopeptide repeats with N- and C-terminal extensions ^15^ (Fig. S1). These repeats share a loosely conserved consensus sequence but strong similarity in amino acid composition ^15^. Notably, the two affected residues lie in the same position in parallel repeats; Arg267 in Repeat 2 and Arg301 in Repeat 3 (Fig. S1). Both Arg267 and Arg301 are completely conserved throughout evolution (Fig. 1A). Arg267 corresponds to tetratricopeptide repeat 2 in the *Saccharomyces cerevisiae* orthologue Clf1p. When a mutant was generated lacking Clf1p repeat 2, it inhibited splicing and caused a temperature-sensitive growth arrest ^16^, supporting that residue fidelity in this repeat is important for function.

The side chains of both Arg267 and Arg301 point towards glutamic acids in the neighbouring helices (Glu232, Glu266), likely indicating the presence of strong ionic interactions (Fig. 1B) ^17^. This region of CRNKL1 is critical for forming hydrophobic patches and a platform for binding by CDC5L, as part of the spliceosome complex states, B^act^ and C. The binding of CDC5L is very likely a prerequisite for the subsequent assembly of the complex containing the proteins PPIL1, U5-40K and parts of RBM22, SKIP and PRP17 (Fig. 1C) ^4^. The substitutions could therefore destabilise the PPIL1 region and interfere with spliceosome maturation. Together, the strong conservation and structural requirements for both affected residues support the prediction of a deleterious consequence when altered by missense variants.

**Figure 2:**
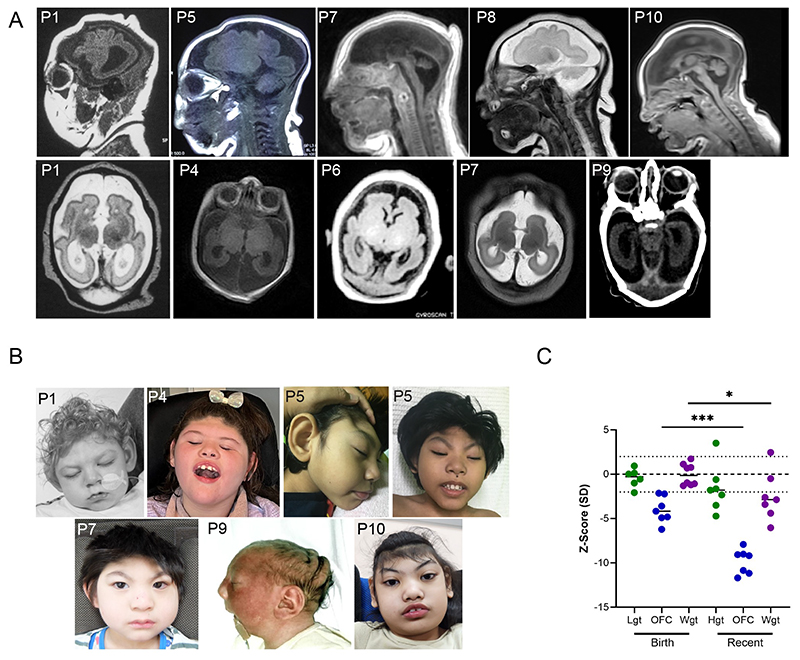
Clinical features of individuals in the *CRNKL1*-associated cohort. **A**. MRI images from different affected individuals illustrating lissencephaly and heavily reduced gyration, prominent CSF-filled spaces in the skull cavity and pontocerebellar hypoplasia. Ages: P1 – 1 week, P4 – 2 weeks. P5 – 3 weeks, P6 – 1 month. P7 – 1 week, P8 – newborn, P9 – 1 week, P10 – 1 month. **B**. Facial images show severe microcephaly altering the skull size and shape, but otherwise no consistent facial dysmorphism. Informed consent or local ethical approval was obtained for all images. Ages: P1 – 1 year, P4 – 9 years, P5 – 5 years 6 months, P7 – 2 years 7 months, P9 - 1 week, P10 – 8 years 9 months. **C**. Anthropometrical measurements from both birth and most recent exam in individuals where measurements are known, showing microcephaly at birth which significantly progresses over time (paired t-test, ***p = 0.0003). Height and weight are not affected at birth, but at most recent exam weight is significantly lower (paired t-test, *p = 0.015).

All affected individuals presented with the same core set of clinical features. Brain MRI scans revealed similar features including a markedly simplified cortical gryal pattern, pontocerebellar hypoplasia, with increased extra-axial spaces (Fig. 2A). Microcephaly is extreme, with pre-natal onset and a progressive decline as individuals age (Fig. 2A, C). Median occipitofrontal circumference (OFC) at birth was -4.17 standard deviations (SD) and -9.18 SD at the most recent exam measurement. While height was not significantly affected, body weight was average at birth but progressively decreased over time (p = 0.015), likely reflecting feeding difficulties and other secondary causes associated with neurodevelopmental syndromes. All individuals have severe neurodevelopmental disability, and most are non-ambulant and non-verbal. Eight out of nine affected individuals have had seizures, with an onset before one year of age. Some individuals showed facial features of arched eyebrows, prominent ears, a short and broad philtrum, wide-spaced teeth and full cheeks, but these features were not universally apparent (Fig. 2B). There were no consistent non-neurological features (Table 1). Four individuals are deceased, one in the first year of life, one at four years, one at five years and the fourth at 16 years of age.

Given the significant genetic evidence implicating alterations to p.Arg267 plus the structurally similar roles in neighbouring α-helices for both p.Arg267 and p.Arg301, alongside a similar and consistent clinical phenotype in all individuals, we conclude that these variants very likely underlie this genetic condition.

To provide functional evidence to further support these findings, we explored the consequences of these variants on spliceosomal function and organism development. We cultured fibroblasts from two individuals (Individuals P4, P5), who both harbour the c.800G>A, p.Arg267His variant. No robust antibodies exist for CRNKL1 and so we were unable to analyse protein levels in these cells, however, through RT-qPCR we observed no difference in *CRNKL1* mRNA transcript levels (Fig. S2A). Transient overexpression of FLAG-CRNKL1 variants in HEK293FT reporter cells demonstrated no obvious reduction in steady-state protein levels (Fig. S2B). In gnomAD v4.1 there are many predicted loss of function variants in *CRNKL1*, and so it is unlikely these identified variants are acting in a direct loss of function manner on the protein.

Microcephaly is commonly caused by a reduction in cell number. Common cellular mechanisms are reduced proliferation, increased apoptosis, or delays in the cell cycle, which could be due to cellular stress ^18^. We measured the growth of proband-derived fibroblasts in culture and observed no difference compared to two control fibroblast lines (Fig. S2C). We used immunocytochemistry to visualise levels of apoptosis (activated caspase-3 staining), DNA damage (γ-H2AX and 53BP1 staining) and mitotic index (phospho-histone H3 staining). In all these assays, we observed no difference between controls and fibroblasts derived from Individuals P4 and P5 (data not shown).

Given the essential role of CRNKL1 in the spliceosome, we explored whether the c.800G>A, p.Arg267His variant caused alterations in gene expression. We sequenced the transcriptome in cell lines derived from Individuals P4 and P5 plus two control fibroblast lines (similar in age and biopsy site, ethnicity similar to P4), using ribo-depleted total RNA. Following sequencing, reads were mapped to the GRCh37 genome using HISAT2 ^19^, and differential gene expression was analysed using DESeq2 ^20^.

RNA-seq confirmed that transcripts from both the wildtype and variant alleles were present in each proband-derived fibroblast cell line (Individual P4: allele depth 32:74, Individual P5: allele depth 24:55) and expression was not significantly different from controls (log_2_ fold change -0.12) (Fig. S3A). When comparing the two sets of cell lines, there was little alteration to global gene expression with only 93 genes showing reduced expression and 25 genes showing increased expression in proband-derived fibroblasts (Fig. S3B) (Table S2). This data suggests that in fibroblasts, the *CRNKL1* variants do not significantly disrupt splicing and gene expression. To validate that significantly altering CRNKL1 activity does have deleterious consequences to cells, we explored the transcriptome of HeLa cells following application of siRNA targeting *CRNKL1*. When *CRNKL1* was heavily depleted in HeLa cells by siRNA (average 80% reduction in reads from *CRNKL1* siRNA-treated samples), there were indeed significant alterations to gene expression, with 2,632 genes showing reduced expression and 2,521 genes showing increased expression in siRNA-treated cells compared to controls (Fig. S3C, D) (Table S3). Many pathways that were upregulated related to RNA processing and translation, and in fact, strikingly, almost all transcripts encoding spliceosomal components were upregulated, suggesting the existence of potentially compensatory mechanisms (Fig. S4A, B). Interestingly, one of the few spliceosomal components downregulated was *WBP4* (MIM:604981) (Table S3), established to be involved in a neurogenetic disorder (MIM:620852) with broader systemic involvement than observed in this cohort ^21^. Cellular pathways that were downregulated included Rho GTPase signalling and senescence-related pathways (Fig. S4C). Depletion of *CRNKL1* caused increased intron retention, with higher ratios of intron retention and increased intron depth in reads from siRNA-treated cells (Fig. S4D, E) (Table S4). Pathways represented in these altered transcripts included RNA processing and cell cycle checkpoints (Fig. S4F).

To analyse developmental consequences of *CRNKL1* variants we used zebrafish as a model (institutional approval for research using zebrafish granted to the University of Otago from the New Zealand Ministry for Primary Industries).

Given the genetic evidence, we hypothesized that the variants act in a gain of function or dominant negative manner, and therefore tested variant effects through microinjecting mRNA encoding different Crnkl1 proteins, using the zebrafish *crnkl1* transcript (NM_200946.1) as the reference: FLAG-Crnkl1-wildtype (referred to as WT), FLAG-Crnkl1-Arg105His - corresponding to p.Arg267His in human CRNKL1 (referred to as p.Arg105His), FLAG-Crnkl1-Arg139Gly - corresponding to p.Arg301Gly in human CRNKL1 (referred to as p.Arg139Gly), FLAG-Crnkl1-STOP (a premature stop codon introduced, to control for RNA toxicity), as well as using dye-only injections, to control for any disruption caused by the physical manipulation. After *in vitro* transcription to generate the mRNA, we undertook a titration experiment to optimise the amount of mRNA to inject. Injection of FLAG-Crnkl1-WT and FLAG-Crnkl1-STOP were tolerated at all injection amounts trialled up to 72 hours post-fertilisation (hpf), indicating that increased presence of wild-type Crnkl1 protein does not hamper zebrafish embryo development. In contrast, injection of FLAG-Crnkl1-Arg105His at 100-300 ng/µL caused significant alterations to development (Fig. S5A), with no survival beyond 24 hpf at 200 or 300 ng mRNA injected. We confirmed by western blotting that similar levels of protein are produced from the WT, p.Arg105His and p.Arg139Gly mRNA injected (Fig S5B).To test whether lower amounts of mRNA caused more subtle physical changes, we used Alcian blue cartilage staining to study craniofacial bone development at 72 hpf in embryos injected with 50 or 75 ng/µL mRNA (Table S5). No abnormalities or differences in measurements were detected, and therefore we proceeded using 100 ng/µL as the injection amount.

**Figure 3:**
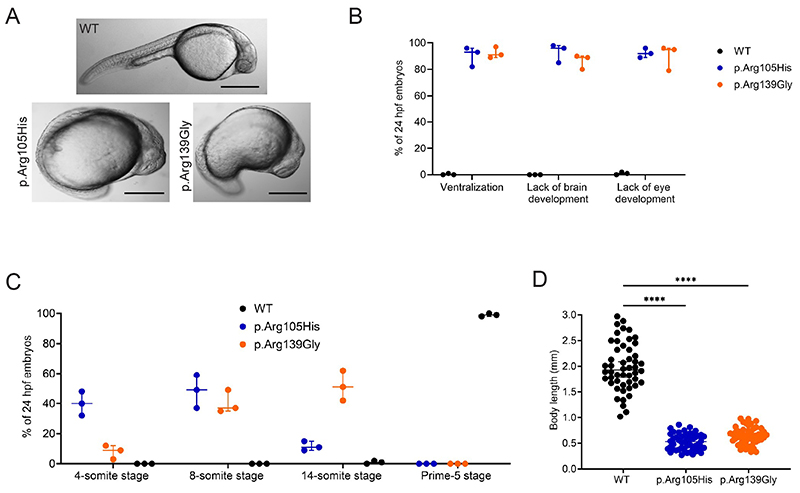
Phenotypic characterisation of a zebrafish *crnkl1* model. **A**. Representative images of 24 hpf embryos injected with either WT crnkl1 or mRNA encoding variant Crnkl1 (p.Arg105His or p.Arg139Gly). Variant Crnkl1 caused significant ventralization, with lack of both brain and eye development (phenotype present in WT: 0%, p.Arg105gHis: 41%, p.Arg139Gly: 47%, n= 3 injection rounds, 50 embryos per injection round). Scale bar = 500 µM. **B**. Quantification of different observed phenotypes in the zebrafish embryos (displayed as a percentage of all embryos showing a phenotype). n= 3 injection rounds, 50 embryos per injection round. **C**. Scoring of developmental stages observed in 24 hpf embryos. n= 3 injection rounds, 50 embryos per injection round. **D**. Measurement of body length, where the p.Arg105His and p.Arg139Gly injected embryos are developmentally delayed and have not straightened out. n= 3 injection rounds, 50 embryos per injection round.

We first analysed 24 hpf embryos for the effects of injecting *crnkl1* mRNA on development. There were no differences in embryo viability (WT: 90%, p.Arg105His: 94%, p.Arg139Gly: 96%). While WT-injected embryos showed no impaired development, 41% of p.Arg105gHis-injected embryos and 47% of p.Arg139Gly-injected embryos showed severe alterations to development (Fig. 3A). In contrast to WT embryos, in the p.Arg105His- and p.Arg139Gly-injected embryos we observed striking ventralization with minimal brain or eye development (Fig. 3B). We compared the characteristic stages of early zebrafish development in the injected embryos, finding both p.Arg105His- and p.Arg139Gly-injected embryos appeared to be delayed in development compared to WT (Fig. 3C), with the majority of embryos classed at the 8-14-somite stage, versus the later Prime-5 stage for almost all WT injected embryos. This delay in development affected embryo size, with the p.Arg105His- and p.Arg139Gly-injected embryos not undergoing straightening of the body axis observed in WT embryos (Fig. 3D). These phenotypes strongly supported an impact on typical zebrafish development caused by p.Arg105His and p.Arg139Gly substitutions in Crnkl1 and align with the severe disruption of brain development observed in the affected individuals.

**Figure 4:**
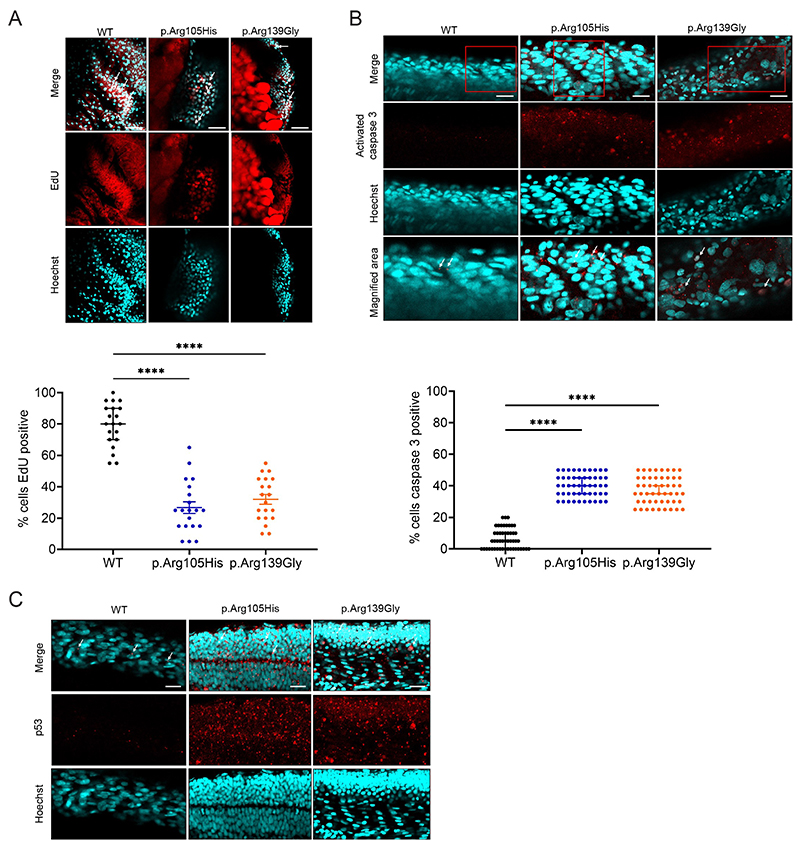
Cellular characterisation of a zebrafish *crnkl1* model. **A**. EdU staining to measure proliferation in WT, pArg105His and p.Arg1139Gly injected embryos. Representative images above, and quantification below. Hoechst staining to indicate DNA. 20 embryos were scored across 3 injection rounds. One-way ANOVA, ****p < <0.0001. Scale bar = 100 µM. **B**. Activated capase3 staining to measure apoptosis in WT, p.Arg105His and p.Arg1139Gly injected embryos. Representative images above, including a magnification outlined by the red box, and quantification below. White arrows indicate cells positive for caspase 3 staining. Hoechst staining to indicate DNA. 50 embryos were scored across 3 injection rounds. One-way ANOVA, ****p < <0.0001. Scale bar = 100 µM. **C**. p53 staining to indicate cellular stress in WT, pArg105His and p.Arg1139Gly injected embryos. Hoechst staining to indicate DNA. Imaging indicated significant cellular stress in the pArg105His and p.Arg1139Gly injected embryos, examples illustrated by white arrows. 20 embryos were imaged across 3 injection rounds, representative images shown. Scale bar = 100 µM.

We next explored what cellular mechanisms could be causing this phenotype, undertaking a similar array of experiments to those performed on the fibroblasts. To examine cell proliferation, we incubated 24 hpf embryos with 10 mM EdU and then processed embryos using the Click-iT reaction cocktail. Nuclear staining was performed with Hoechst 33342 in accordance with the manufacturer’s instructions. EdU-positive cells were quantified using ImageJ software. In WT embryos, an average of 85% of cells showed EdU incorporation (Fig. 4A). In the p.Arg105His- and p.Arg139Gly-injected embryos, only an average of 30% of cells for p.Arg105His and 35% of cells for p.Arg139Gly showed EdU incorporation (p < 0.0001, 20 embryos across 3 injection rounds) (Fig. 4A), indicating reduced cell proliferation in the p.Arg105His- and p.Arg139Gly-injected embryos. To measure apoptosis, we undertook whole-mount immunohistochemistry staining for activated caspase 3 (Fig. 4B). While WT injected embryos showed minimal levels of apoptosis, both p.Arg105His- and p.Arg139Gly-injected embryos showed 38% and 40% cells positive for activated caspase 3 staining, respectively (p < 0.0001, n = 3 injections, 50 embryos across 3 injection rounds). Finally, we examined p53 staining using whole-mount immunohistochemistry as an indicator of cell stress. As expected, a low level of p53 staining was observed in WT injected embryos. In contrast, p.Arg105His- and p.Arg139Gly-injected embryos showed a striking level of p53 staining (Fig. 4C). From these experiments, our results indicate that altered spliceosomal activity caused by mutant Crnkl1 elevates cellular stress which impedes normal levels of cell proliferation and apoptosis.

**Figure 5:**
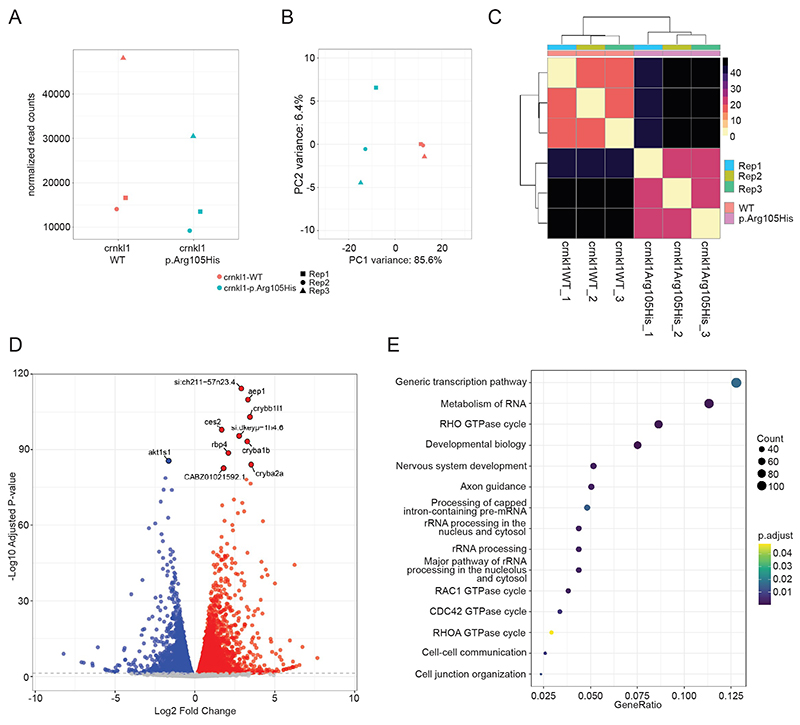
Analysis of RNA-seq data obtained from zebrafish embryos. **A**. Normalized read counts for *crnkl1* transcripts in three replicates of WT and p.Arg105His embryos (each replicate indicated by different symbols). Reads from only the introduced mRNA and not endogenous *crnkl1* were identified using the FLAG tag transcript sequence. **B**. PCA plot of the six RNA-seq samples, showing that the most variance in the samples is between the WT and p.Arg105His groupings (separation on PC1), confirming the variant Crnkl1 is driving molecular differences. **C**. Correlation matrix heatmap between the six samples, showing that the three replicates (1-3) from each genotype group separately and with greater similarity versus the other genotype. **D**. Statistically significant alterations to gene expression identified by DESeq2 indicated by red and blue dots (5% false discovery rate). 4614 genes have reduced expression in p.Arg105His compared to WT injected embryos and 4715 genes have increased expression in p.Arg105His compared to WT injected embryos. **E**. Pathway over representation analysis using ReactomePA of genes identified as showing statistically significant reductions in expression.

To further understand the cellular changes caused by the variants, we undertook transcriptomic analysis. We pooled 25 24 hpf embryos from either WT or p.Arg105His-injected embryos, with three independent injection rounds as replicates and followed an established RNA-seq analysis pipeline (see Online methods). We first confirmed that there were similar normalized read counts from the injected mRNA across all samples, using the FLAG sequence as an identifier (Fig. 5A). A PCA plot and heatmap supported the separation of the WT versus p.Arg105His samples, confirming that the biological differences are more significant than any noise or potential batch effects of different injection rounds (Fig. 5B, C). Analysis of differential gene expression showed the p.Arg105His-injected embryos showed a significant number of differentially expressed genes (threshold p-value < 0.05); 4614 genes showing reduced expression and 4715 genes showing increased expression in the p.Arg105His-injected embryos compared to WT, supporting widespread changes in gene regulation (Fig. 5D) (Table S6). To understand global patterns, we used ReactomePA to assess biological pathways ^22^. We focused on downregulated pathways, as these are more likely to represent direct consequences of inaccurate splicing (Fig. 5E). Down-regulated pathways included nervous system development and axon guidance as well as a number of RNA-related processes, including RNA metabolism and rRNA processing. Several of these identified pathways (RNA metabolism, axon development, ribosome/rRNA processing) were similarly enriched in the splicing pathway analysis of RNA-seq from the *Ppil1* knockin mice brains, where human *PPIL1* variants cause the same phenotype ^10^. RHO, RAC1 and CDC42 GTPase pathways were downregulated in our analysis. These GTPases are essential for migration at various points during neurogenesis ^23^, with deficiency of Rac1 or Rho known to impair brain development and cause microcephaly or holoprosencephaly in mouse models ^24,25^. Together, our results show disrupted pathways relevant to brain development, and a number overlapping with those identified in studies of *PPIL1*.

In summary, we have identified a cohort of individuals with de novo missense variants in *CRNKL1*. One amino acid (p.Arg267) is particularly impacted by recurrent variants, while the other impacted residue (p.Arg301) lies in a parallel position in the neighbouring α-helix, suggesting that positional effects impact on CRNKL1 and spliceosomal function. All affected individuals share a common neurological phenotype of severe microcephaly and structural brain anomalies. Studying the variants using a zebrafish model revealed significant effects on cellular stress, proliferation and apoptosis – common pathways observed in other microcephaly disorders. Together, our genetic and developmental evidence highly supports specific variants in *CRNKL1* causing a neurogenetic disorder of microcephaly and pontocerebellar hypoplasia.

Recently, biallelic variants in *PPIL1* and *PRP17* have been demonstrated to cause a phenotypically very similar disorder of microcephaly with pontocerebellar hypoplasia ^10^, with little impact on other body systems. Many of these individuals also had epilepsy and severe impairment of cognition and development. Compared to the individuals described here, the *PPIL1*/*PRP17*-associated cohort had milder microcephaly, and structural anomalies were not as severe on brain imaging. While the *PPIL1*/*PRP17*-associated disorder was described as neurodegenerative, we do not observe this as a strong feature in our cohort. While four individuals are deceased, several individuals with *CRNKL1* variants have reached adulthood. Following the cohort for longer and identifying further affected individuals will provide more information to help compare with the PPIL1/PRP17-related neurodegeneration. CRNKL1, along with PPIL1 and PRP17, is an associated member of the PRP19 subcomplex within the spliceosome. Of the other subcomplex members, recently, de novo variants have been described in *PRPF19* (MIM: 608330), which encodes PRP19 ^26^. While these individuals present with a neurodevelopmental disorder, no structural brain abnormalities were reported in the cohort. Facial dysmorphism was a consistent feature (5/5) and vision abnormalities were common (3/5 individuals). This clinical data suggests that while PRP19 is part of a subcomplex with CRNKL1, PPIL1 and PRP17, genetic alterations of these components can give rise to different developmental effects. We have identified a hotspot for *de novo* missense variants in *CRNKL1*, with the high recurrence of two variants at the same amino acid and presence of pLoF variants in gnomAD both arguing against haploinsufficiency or direct loss of function. For *PPlL1* and *PRP17*, individuals were biallelic for hypomorphic loss of function variants, with missense variants decreasing protein stability or impeding protein interactions ^10^. Our separate analyses of the Hela siRNA-treated and zebrafish RNA-seq identified similar biological pathways (RNA processing and RHO GTPases) that were altered by either reducing CRNKL1 expression or injecting mRNA encoding the variant crnkl1-p.Arg105His. Our intron retention analysis from the HeLa siRNA-treated RNA-seq identified a large number of altered splicing events, confirming that disrupting CRNKL1 function impairs accurate splicing. Bringing all these observations together, we therefore speculate that the CRNKL1 substitutions act in a dominant-negative manner to ultimately reduce CRNKL1 function, impairing accurate and efficient splicing. Further experiments to examine the consequences of the missense variants on the structure and the impact on splicing mechanics are needed to confirm the molecular mechanisms underlying this disorder.

RNA-seq analysis comparing WT and p.Arg105His injected zebrafish embryos identified fundamental processes for cell survival and development as being down-regulated, including transcription, RNA metabolism and the GTPase cycle. Interestingly, many of these were also found to be affected in splicing analysis in the *Ppil1* knock-in mouse model, suggesting common pathways could be affected ^10^. Splicing analysis was not robust enough for interpretation of the RNA-seq data from our zebrafish model (likely due to insufficient read depth), but splicing analysis from the *Ppil1* knock-in mouse showed disrupted alternative splicing, with short and high-GC content introns particularly targeted ^10^. Given the close localisation within the spliceosome and the common human phenotype, it would not be surprising if *crnkl1* variants have a similar impact on alternative splicing.

It is not clear why individuals with variants in *CRNKL1* (and *PPIL1* and *PRP17*) show primarily a neurological phenotype. The majority of spliceosomal proteins are expressed ubiquitously, but genetically associated tissue-specific phenotypes are surprisingly common ^5^. For some of these, it appears that mis-splicing of a crucial gene leads to the specific phenotype, but no clear candidates emerge from the transcriptomic analysis undertaken here using the zebrafish model. Analysing the transcriptome of just the head/brain region may provide more insight, though we note our whole-body RNA-seq approach generated similar results to the *Ppil1* mouse model, where only mRNA from brain tissue was sequenced ^10^. A specific role for crnkl1 in *Drosophila* has been described to help control glial cell maturation, further pointing to its requirement in neurogenesis ^27^. Within the spliceosome, CRNKL1, PPIL1 and PRP17 proteins are located close together, suggesting a common dysregulated function of this area of the spliceosome could be causing this neurological phenotype. PPIL1 and PRP17 bind to form an isomerase-substrate interaction, but this isomerization of PRP17 by PPIL1 is not critical for function. The precise impact of *PPIL1, PRP17* and now *CRNKL1* variants on the protein mechanics of the spliceosome and splicing pathway remains to be uncovered.

## Supplementary Material

Supplemental Figures and Methods

Table S1

Table S2

Table S3

Table S4

Table S6

## Figures and Tables

**Table 1:** Genetic and clinical summary of individuals with *CRNKL1* variants.

Individual	P1	P2	P3	P4	P5	P6	P7	P8	P9	P10
Country / Region of origin	Germany	N European	USA	New Zealand	Malaysia	Belgium	Japan	UK	Estonia	Malaysia
Sex	male	female	female	female	male	female	male	female	male	female
Variant	c.799C>T, p.Arg267Cys	c.799C>T, p.Arg267Cys	c.799C>T, p.Arg267Cys	c.800G>A, p.Arg267His	c.800G>A, p.Arg267His	c.800G>A, p.Arg267His	c.800G>A, p.Arg267His	c.800G>A, p.Arg267His	c.800G>A, p.Arg267His	c.901C>G, p.Arg301Gly
Inheritance	*de novo*	*de novo*	na	*de novo*	*de novo*	*de novo*	*de novo*	*de novo*	na	*de novo*
Microcephaly	-9.01 SD*	na	na	-7.9 SD	-9.2 SD	-11.2 SD	-11.7 SD	-9.1 SD	-4.9 SD (birth)	-10.8 SD
Simplified gyration	+	+	+	+	+	+	+	+	+	+
Pontocerebellar hypoplasia	+	+	+	+	+	na	+	+	+	+
Seizures	+	na	+	+	+	+	na	+	+	+
Intellectual disability	++	na	++	++	++	++	++	++	na	++

na, not available. *SD – standard deviation, where -2 is the lower end of typical variation in head size in the population. RefSeq: NM_016652.6

## Data Availability

The HeLa siRNA and zebrafish RNA-seq datasets are available at NCBI GEO under accession numbers GSE294227 and GSE294226.
